# Fluctuations in emergency department visits related to acute otitis media are associated with extreme meteorological conditions

**DOI:** 10.3389/fpubh.2023.1153111

**Published:** 2023-06-01

**Authors:** Michael Nieratschker, Markus Haas, Mateo Lucic, Franziska Pichler, Faris F. Brkic, Thomas Parzefall, Dominik Riss, David T. Liu

**Affiliations:** Department of Otorhinolaryngology, Head and Neck Surgery, Medical University of Vienna, Vienna, Austria

**Keywords:** acute otitis media, ear, emergency service, epidemiology, weather

## Abstract

**Background:**

Climate change has been associated with a higher frequency of extreme weather events, resulting in an overall increase in morbidity and mortality. Acute otitis media (AOM) is one of the most common otolaryngological infections and accounts for 1.5% of emergency department visits. This study aimed to identify associations between extreme weather events and the immediate and delayed risks for AOM-related emergency department visits (EV).

**Methods:**

A total of 1,465 AOM-related EVs were identified in the Vienna General Hospital between 2015 and 2018. A distributed lag non-linear model was applied to evaluate the relationship between extreme weather conditions and the total number of AOM-related EVs per day. The relative risk (RR) and cumulative RR (cRR) of single-day events and extended weather events over three days were analyzed over a lag period of 14 days.

**Results:**

AOM-related EVs showed a pronounced seasonality, with the highest occurrence during winter. Single-day weather events affected AOM-related EVs only at high relative humidity. Prolonged extreme weather conditions over three days, however, significantly increased the cRR for AOM-related EVs to 3.15 [1.26–7.88; *p* = 0.014] and 2.14 [1.14–4.04; *p* = 0.018] at mean temperatures of −4°C (1^st^-percentile - p_1_) and 0°C (p_5_) on the same day. Relative humidity of 37% (p_1_) decreased RR to 0.94 [0.88–0.99; *p* = 0.032] on day 7, while extremely high humidity of 89% (p_99_) led to an increased cRR of 1.43 [1.03–2.00; *p* = 0.034] on day 7. Heavy prolonged precipitation of 24mm (p_95_) reduced cRR beginning day 4 up until day 14 to 0.52 [0.31–0.86; *p* = 0.012]. Prolonged low atmospheric pressure events of 985hPa (p_5_) reduced the RR to 0.95 [0.91–1.00; *p* = 0.03], whereas extremely high atmospheric pressure events of 1013hPa (p_99_) increased the RR to 1.11 [1.03–1.20; *p* = 0.008]. Extremely low wind speeds significantly diminished the RR of AOM-related EVs.

**Conclusions:**

While single-day extreme weather events had little impact on the occurrence of AOM-related EVs, extended periods of extreme temperatures, relative humidity, precipitation, wind speeds and atmospheric pressure significantly impacted the RR for AOM-related EVs. These findings could help improve healthcare resource allocation in similar climates and aid in educating patients about the role of environmental factors in AOM.

## Introduction

Extreme weather events are one of the main consequences of the global rise in temperature associated with climate change, which negatively impacts various health conditions such as cardiovascular risk, pneumonia, and infectious disease transmission (1–3). Acute otitis media (AOM) is one of the most frequent otolaryngologic diagnoses among emergency department visits (EV) (4, 5), and represents a significant burden to healthcare systems worldwide (6, 7). Identifying environmental factors and the impact of extreme weather events on the risk for AOM-related EVs might facilitate optimal allocation of healthcare resources and improve overall patient outcomes. Previous studies have shown an effect of temperature and relative humidity on AOM-related EVs (8, 9). However, it is currently unclear how extreme weather conditions influence AOM-related EVs over time.

AOM is one of the most common infections in otolaryngological and pediatric practice and accounts for 1.5% of all emergency department visits (10). Etiologically, AOM is a multifactorial disease in which infectious, allergic, and environmental causes contribute to the onset of the disease (11). AOM is routinely preceded by viral upper respiratory infections (URI), most commonly respiratory syncytial virus (RSV), coronaviruses, influenza viruses, and adenoviruses (12, 13). Inflammation causes dysfunction of the Eustachian tube (ETD), resulting in negative pressure in the middle ear, which facilitates the influx of viruses and bacteria into the middle ear (14). This potentially induces a secondary bacterial infection by the naturally residing pathogens of the nasopharynx, *S. pneumoniae, H. influenzae*, and *M. catarrhalis* (11). Clinically, most common AOM symptoms are otalgia, dull and diminished hearing, and may be accompanied by otic discharge.

The primary age of onset of AOM is in newborns and children between 4 months and 3 years. It is assumed that an anatomically shorter and more horizontally oriented eustachian tube promotes easier migration of pathogens into the middle ear (15). Previous studies on the influence of meteorologic factors on AOM occurrence have, therefore, focused on the pediatric population, neglecting AOM-related EVs in adults (8, 9, 16). However, a study by Ren et al. showed that of all patients who developed complications related to AOM, 76% occurred in adults (10). Complications result from pus formation in the middle ear and a subsequent spread to adjacent anatomic structures, manifesting as bacterial meningitis, venous sinus thrombosis, labyrinthitis, mastoiditis, and facial nerve paralysis (10).

Environmentally, AOM has been shown to be more frequent during winter months (8, 9). Meteorological factors such as temperature, humidity, and atmospheric pressure have been shown to influence AOM-related EVs, with an increased relative risk observed at low temperatures and relative humidity as well as during high atmospheric pressure events (8, 17, 18). Viral URI, which are associated with a significant number of AOM cases, are similarly correlated with temperature and humidity (19, 20). To the best of our knowledge, currently, no data exists on the effects of extreme weather events on AOM-related EVs in a predominantly adult patient population. Furthermore, previous studies have focused on the same-day effects on disease occurrence, which might simplify weather's effects on the occurrence of disease (18, 21).

Therefore, this study aimed to evaluate the immediate and delayed effects of extreme weather events on the occurrence of AOM-related EVs in a tertiary hospital in Vienna, Austria. We utilized a distributed lag non-linear model (DLNM) and analyzed single-day and prolonged effects of mean temperature, relative humidity, mean wind speed, precipitation, and atmospheric pressure over 14 days.

## Methods

### Study population and meteorological data

In this study, all emergency department visits related to AOM, from January 1, 2015, to December 31, 2018, were extracted from the digital medical record system of the Vienna General Hospital. Basic demographics such as age, sex, visit date, referral status, and clinical information such as affected ear and complications were evaluated. This study was approved by the ethics committee of the Medical University of Vienna (#2136/2019 and #2121/2019).

Meteorological data from January 1, 2015, to December 31, 2018, was provided by Austria's national meteorological and geophysical service – “Zentralanstalt für Meteorologie und Geodynamik” (ZAMG) and included daily mean temperature, relative humidity, precipitation, mean wind speed, and atmospheric pressure. All weather data was collected at the central Vienna weather station (latitude: 48.198°; longitude: 16.3669°) at an elevation of 177 m above sea level. Due to the homogeneity of the Viennese weather, a single weather station was chosen to represent whole federal state of Vienna with a catchment area of 414.6 km^2^ and a population of 1.9 M (22, 23).

### Statistical analysis

Immediate and delayed effects of extreme weather on the frequency of AOM-related EVs were assessed by fitting a DLNM to the dataset (24). DLNMs have previously been utilized to describe the delayed effects of air pollution and meteorological events on the occurrence of AOM and general morbidity and mortality (18, 25). In this study, the following independent variables were defined: mean temperature, relative humidity, precipitation, mean wind speed, and atmospheric pressure. For each meteorological variable, a separate model was calculated. The response variable was defined as the number of daily EVs related to AOM. Two bases of each model were chosen to describe the relationship between weather data and lag-days (lags). Lags are the days following the initial exposure to a weather condition. For the daily weather variables, natural cubic splines with five degrees of freedom (df) at equally spaced percentiles and equal intervals on the logarithmic scale were selected (26). The maximum lag was set to 14 days to account for potential harvesting effects (27).

To control for long-time trends and seasonality, natural cubic splines of time with seven df per year were used. Indicator variables for each day of the week were added to account for varying demand during the week, such as closed private practices on weekends. Additionally, public holidays were controlled using a dummy variable, as an increase in EVs is expected on these days. On public holidays, hospital outpatient departments and private practices such as general practitioners (GP) and otorhinolaryngologists are closed in Austria, making emergency departments the only point of contact for acute medical conditions, possibly resulting in increased visitation rates. Since holidays may also fall on weekend days which might reduce their effect on EVs, an interaction between the day of the week and the public holiday dummy was added. Coefficient estimates indicate fluctuating EV rates between weekdays and a significant increase of EVs on weekends and public holidays. Furthermore, the negative interaction between weekend days and public holidays was statistically significant. Therefore, EV rates are not expected to increase when public holidays occur on weekend days. An additional dummy variable was added to control for a major policy change in the EVs admission practice in December 2016, at which a screening station of GPs was implemented prior to the emergency department. This procedural change did not significantly alter EVs for AOM.

Extreme weather events were defined as each independent weather variable's 1st, 5th, 95th, and 99th percentile. The relative risk (RR) of AOM-related EVs was calculated for each lag-day of each extreme weather event using the median of each weather variable as a reference. The cumulative RR (cRR) is defined as the risk of an EV after an extreme weather condition compared to the risk of an EV at the median value of that weather condition within the stated period, calculated by cumulating the RR from lag0 up to lag14. RR values, including confidence interval and p-values, were extracted for lag0, lag1, lag4, lag7, and lag14 (**Table 2**) and in case of cRR for lag0–1, lag0–4, lag0–7, and lag0–14 (Supplementary Table 1). Additionally, we accounted for sustained extreme weather conditions by calculating the RR and cRR for events lasting three days. The three-day mean was used for mean temperature, relative humidity, mean wind speed, and atmospheric pressure. For precipitation, the sum over three days was used. Extended events were calculated using a rolling time window in which the total number of AOM-related EVs in the previous three days served as the outcome variable. The values for RR (**Table 3**) and cRR (Supplementary Table 2) are stated similarly to those of the one-day model.

To further account for seasonal differences in the effects of extreme weather events, a stratified analysis was conducted by using a separate summer and winter model. The summer model included a time span from 1 of April to 30 of September, while the winter model was calculated from 1 of October to 31 of March of the following year. Similar to the whole-year model, RR for the 1st, 5th, 95th and 99th percentile of each weather variable was calculated for the summer and winter model, respectively (Supplementary Tables 3–6).

Statistical testing, model fitting and plotting of contour plots were performed using R software (version 4.1.3) (28). DLNMs were fitted using the “dlnm” package (29). Contour plots were drawn using the “ggplot2” package. Graphs were plotted using GraphPad Prism 9.5 software. Data is presented as RR or cRR [95% confidence interval (CI)].

## Results

### Study population and weather

**Figure 1:**
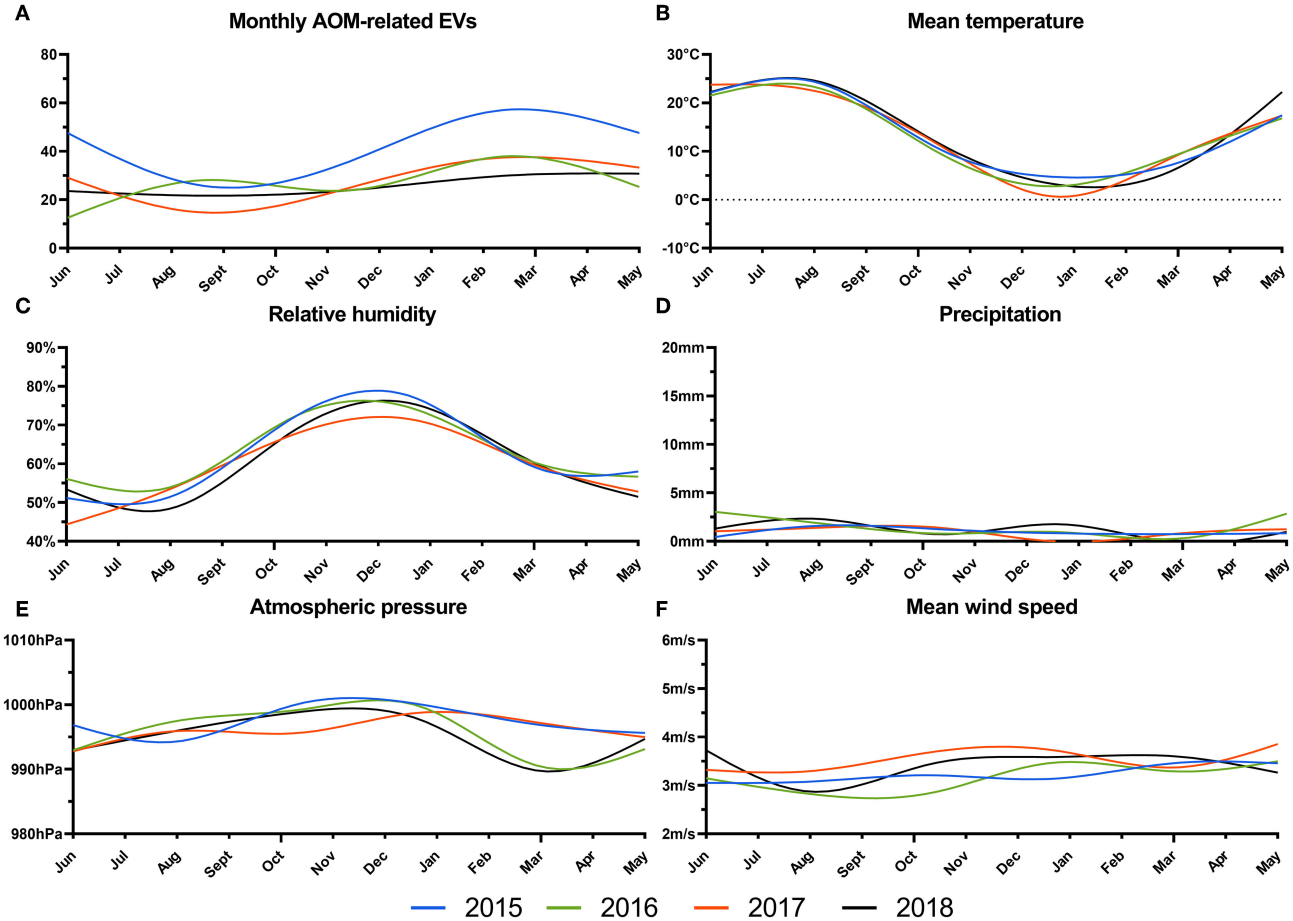
Total number of acute otitis media-related EVs **(A)**, and daily mean temperature in °C **(B)**, relative humidity in % **(C)**, mean precipitation in mm **(D)**, atmospheric pressure in hPa **(E)** and mean wind speed in m/s **(F)** from 2015 to 2018 are plotted as smoothed cubic splines.

Overall, at the Vienna General Hospital, Austria, 1465 AOM-related emergency visits occurred between January 1, 2015, and December 31, 2018. Patient characteristics of the patient cohort are described in detail in Table 1. Of the patient cohort, 79.7% were over 18 years of age. On average, more than one AOM-related EV occurred daily. AOM-related EVs followed a clear seasonal trend in 2015, 2016 and 2017, with the highest rates in the winter months between January and March and the lowest occurrence in August and September (Figure 1A). The mean temperature and relative humidity followed an expected seasonal trend with the lowest temperatures and highest humidity in winter months (Figures 1B, C). However, precipitation, atmospheric pressure and mean wind speed did not show a relevant seasonal pattern (Figures 1D–F).

**Table 1:** Patient characteristics of the study cohort presenting with acute otitis media in the emergency department.

**Characteristics**		**Study cohort, *N* (%)**
**Total**		1,465 (100%)
**Age at presentation**		
	0–17	297 (20.3%)
	18–45	916 (62.5%)
	46–64	197 (13.4%)
	65+	55 (3.8%)
**Gender**		
	Male	731 (49.9%)
	Female	734 (50.1%)
**Affected Ear**		
	Left	682 (46.5%)
	Right	680 (46.4%)
	Bilateral	103 (7%)
**Complications**		
	TM perforation	28 (1.9%)
	Mastoiditis	83 (5.7%)
	None	1,354 (92.4%)
**Referral from external provider**		
	Yes	236 (16.1%)
	No	1,226 (83.7%)
	Unknown	3 (0.2%)

### Mean temperature

**Figure 2:**
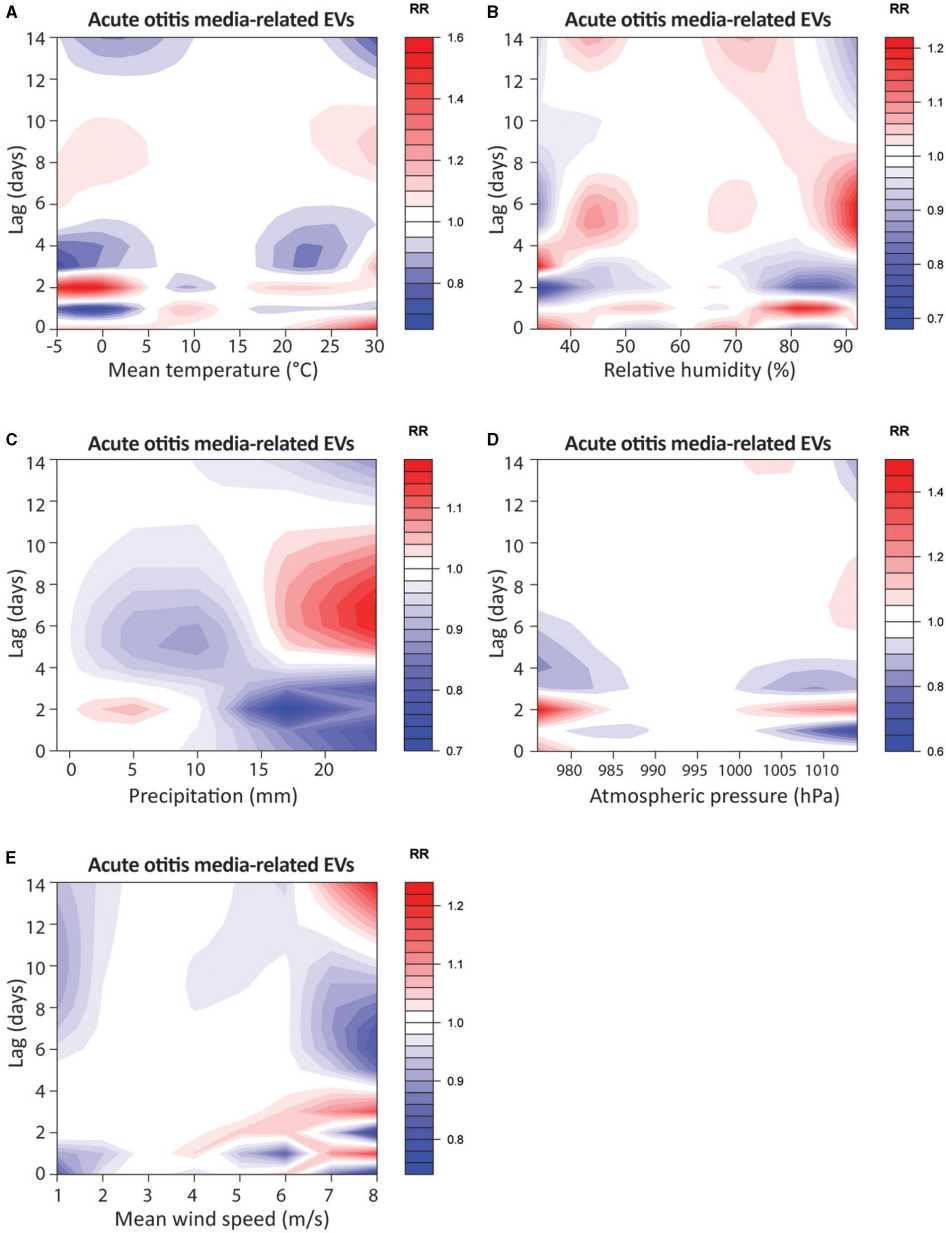
Contour-plots of relative risk for acute otitis media-related EVs of mean temperature in °C **(A)**, relative humidity in % **(B)**, precipitation in mm **(C)**, atmospheric pressure in hPa **(D)** and mean wind speed (m/s) **(E)** from lag0 to lag14.

In the first step, we evaluated the effect of mean temperature on AOM-related EVs (Figure 2A). Low temperatures resulted in a RR ranging from 1.13 [0.49–2.61] at −5°C and 1.15 [0.70–1.90] at 0°C on the same day, up to 0.96 [0.73–1.26] and 0.85 [0.69–1.03] 14 days after the weather event. Extremely high temperatures of 27°C and 30°C resulted in RRs ranging from 1.33 [0.77–2.30] and 1.52 [0.74–3.11] on the same day to 0.86 [0.70–1.06] and 0.76 [0.58–1.01] 14 days following the weather event. Overall, neither extremely low temperatures of −5°C and 0°C (p_1_ and p_5_) nor extremely high temperatures of 27°C and 30°C (p_95_ and p_99_) showed a significant change in the RR for AOM-related EVs (*p* > 0.05, Table 2). Similarly, no significant difference in cRR was noticed at any lag interval over 14 days at low and high temperatures (Supplementary Table 1).

**Table 2:** Relative risk for acute otitis media-related EVs under extreme weather conditions (1st, 5th, 95th, and 99th percentile) compared to median conditions.

**Relative risk (RR) of AOM–related EVs**				
**Mean temperature**	−**5** °**C (p**_1_**)**	**0** °**C (p**_5_**)**	**27** °**C (p**_95_**)**	**30** °**C (p**_99_**)**
Lag0	1.13 [0.49–2.61]; *p =* 0.78	1.15 [0.7–1.9]; *p =* 0.58	1.33 [0.77–2.3]; *p =* 0.306	1.52 [0.74–3.11]; *p =* 0.252
Lag1	0.78 [0.23–2.62]; *p =* 0.688	0.68 [0.34–1.35]; *p =* 0.268	0.92 [0.44–1.95]; *p =* 0.828	0.89 [0.33–2.41]; *p =* 0.818
Lag4	0.81 [0.59–1.1]; *p =* 0.18	0.85 [0.7–1.03]; *p =* 0.102	0.91 [0.74–1.11]; *p =* 0.344	1.04 [0.79–1.36]; *p =* 0.804
Lag7	1.08 [0.91–1.27]; *p =* 0.366	1.05 [0.94–1.17]; *p =* 0.362	1.02 [0.91–1.15]; *p =* 0.694	1.05 [0.9–1.21]; *p =* 0.54
Lag14	0.96 [0.73–1.26]; *p =* 0.766	0.85 [0.69–1.03]; *p =* 0.098	0.86 [0.7–1.06]; *p =* 0.162	0.76 [0.58–1.01]; *p =* 0.062
**Relative humidity**	**34 % (p**_**1**_ **)**	**39 % (p**_**5**_ **)**	**86 % (p**_**95**_ **)**	**92 % (p**_**99**_ **)**
Lag0	1.14 [0.79–1.65]; *p =* 0.484	1.09 [0.86–1.37]; *p =* 0.488	0.88 [0.7–1.11]; *p =* 0.28	1.03 [0.71–1.5]; *p =* 0.878
Lag1	0.99 [0.64–1.53]; *p =* 0.972	1 [0.77–1.3]; *p =* 0.998	1.16 [0.91–1.49]; *p =* 0.234	0.99 [0.65–1.5]; *p =* 0.956
Lag4	1.2 [0.93–1.55]; *p =* 0.156	1.02 [0.85–1.22]; *p =* 0.864	0.92 [0.76–1.11]; *p =* 0.394	0.93 [0.68–1.25]; *p =* 0.612
Lag7	0.89 [0.78–1]; *p =* 0.058	0.99 [0.91–1.07]; *p =* 0.8	1.05 [0.98–1.14]; *p =* 0.188	**1.14 [1.01–1.28];** ***p** **=*** **0.028**
Lag14	0.94 [0.75–1.17]; *p =* 0.568	1.04 [0.9–1.2]; *p =* 0.58	0.96 [0.84–1.1]; *p =* 0.562	0.88 [0.71–1.1]; *p =* 0.268
**Precipitation**	–	–	**10 mm (p**_**95**_ **)**	**24 mm (p**_**99**_ **)**
Lag0	–	–	0.97 [0.79–1.21]; *p =* 0.804	0.8 [0.47–1.36]; *p =* 0.408
Lag1	–	–	0.98 [0.79–1.22]; *p =* 0.844	0.79 [0.45–1.37]; *p =* 0.402
Lag4	–	–	0.91 [0.82–1.02]; *p =* 0.118	0.95 [0.7–1.31]; *p =* 0.77
Lag7	–	–	0.92 [0.84–1]; *p =* 0.058	1.16 [0.91–1.48]; *p =* 0.222
Lag14	–	–	0.98 [0.85–1.13]; *p =* 0.772	0.88 [0.6–1.29]; *p =* 0.51
**Mean wind speed**	**1.0 m/s (p**_**1**_ **)**	**1.5 m/s (p**_**5**_ **)**	**5.9 m/s (p**_**95**_ **)**	**7.5 m/s (p**_**99**_ **)**
Lag0	0.84 [0.64–1.11]; *p =* 0.218	0.97 [0.89–1.06]; *p =* 0.536	1.06 [0.84–1.33]; *p =* 0.638	0.75 [0.39–1.45]; *p =* 0.396
Lag1	0.91 [0.69–1.19]; *p =* 0.482	0.94 [0.86–1.02]; *p =* 0.13	0.84 [0.66–1.07]; *p =* 0.168	1.17 [0.6–2.27]; *p =* 0.642
Lag4	1 [0.86–1.17]; *p =* 0.988	1 [0.96–1.05]; *p =* 0.956	1.01 [0.89–1.16]; *p =* 0.846	1.01 [0.73–1.41]; *p =* 0.936
Lag7	0.94 [0.82–1.07]; *p =* 0.342	0.99 [0.95–1.03]; *p =* 0.496	0.99 [0.89–1.1]; *p =* 0.812	0.83 [0.63–1.09]; *p =* 0.176
Lag14	0.93 [0.77–1.13]; *p =* 0.472	0.97 [0.91–1.02]; *p =* 0.246	0.95 [0.8–1.14]; *p =* 0.6	1.22 [0.82–1.82]; *p =* 0.326
**Atmospheric pressure**	**976 hPa (p**_**1**_ **)**	**983 hPa (p**_**5**_ **)**	**1009 hPa (p**_**95**_ **)**	**1014 hPa (p**_**99**_ **)**
Lag0	1.19 [0.77–1.84]; *p =* 0.446	1.02 [0.78–1.34]; *p =* 0.868	1.06 [0.8–1.41]; *p =* 0.674	1.06 [0.62–1.8]; *p =* 0.836
Lag1	1 [0.6–1.66]; *p =* 1	0.91 [0.66–1.25]; *p =* 0.558	0.78 [0.54–1.12]; *p =* 0.182	0.64 [0.33–1.25]; *p =* 0.19
Lag4	0.83 [0.66–1.05]; *p =* 0.118	0.91 [0.81–1.03]; *p =* 0.134	0.9 [0.81–1.01]; *p =* 0.078	0.92 [0.77–1.1]; *p =* 0.356
Lag7	0.96 [0.82–1.13]; *p =* 0.63	0.98 [0.91–1.07]; *p =* 0.72	1.04 [0.97–1.11]; *p =* 0.308	1.08 [0.96–1.22]; *p =* 0.19
Lag14	0.96 [0.76–1.22]; *p =* 0.754	0.98 [0.85–1.13]; *p =* 0.744	1.01 [0.89–1.14]; *p =* 0.882	0.85 [0.69–1.05]; *p =* 0.136

Brackets contain confidence intervals (95%); Significant results (p ≤ 0.05) are highlighted.

**Figure 3:**
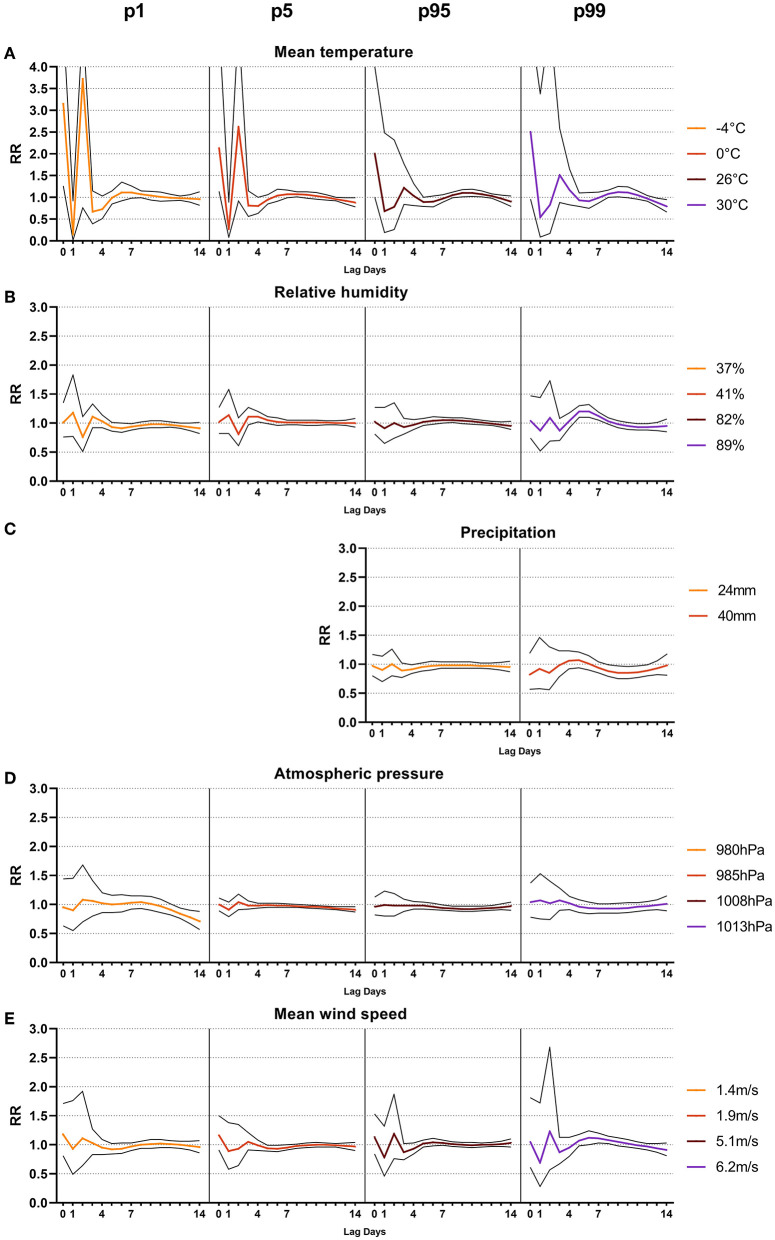
Line-plots (confidence interval 95%) of relative risk (RR) after sustained extreme weather events (1st, 5th, 95th and 99th percentile) of three days from lag0 to lag14 of mean temperature in °C **(A)**, mean relative humidity in % **(B)**, sum of precipitation in mm **(C)**, mean atmospheric pressure in hPa **(D)** and mean wind speed in m/s **(E)**.

At prolonged low temperatures of −4°C and 0°C (p_1_ and p_5_) over three days, a significant increase in the RR on the same day, with 3.15 [1.26–7.88; *p* = 0.014] and 2.14 [1.14–4.04; *p* = 0.018], respectively, was observed (Figure 3A). One day after prolonged low temperatures, the RR was significantly decreased to 0.15 [0.02–0.92; *p* = 0.04] and 0.27 [0.08–0.89; *p* = 0.03] at −4°C and 0°C (p_1_ and p_5_), respectively. Subsequently, the RR of AOM-related EVs at −4°C (p_1_) ranged from 0.73 from lag-day 4 to 0.96 at lag-day 14, without being significantly different (*p* > 0.05). At 0°C, the RR ranged from 0.80 at lag-day 14 (*p* > 0.05) to a significantly decreased RR of 0.88 [0.78–0.99] (*p* = 0.036) at lag-day 14. Similarly, at prolonged high temperatures events of 26°C and 30°C (p_95_ and p_99_) over three days, a non-significant increase in RR with 2.00 [1.00–4.00] and 2.51 [0.96–6.56] on day 0 was observed (*p* > 0.05). On the other time points, no significant difference was observed at any timepoints (Table 3). In addition to the increased cRR for AOM-related EVs after a cold spell of 3 days, a significant increase in cRR to 2.00 (*p* = 0.002) and 1.69 (*p* = 0.014) within 4 to 7 days after prolonged extremely high temperatures of 30°C was observed (Supplementary Table 2).

**Table 3:** Relative risk for acute otitis media-related EVs after prolonged extreme weather conditions compared to median conditions.

**Relative risk (RR) of AOM-related EVs**				
**after prolonged extreme weather conditions over 3 days**				
**Mean temperature**	−**4** °**C (p**_1_**)**	**0** °**C (p**_5_**)**	**26** °**C (p**_95_**)**	**30** °**C (p**_99_**)**
**over 3 days (mean)**				
Lag0	**3.15 [1.26–7.88];** ***p** **=*** **0.014**	**2.14 [1.14–4.04];** ***p** **=*** **0.018**	2 [1–4]; *p =* 0.052	2.51 [0.96–6.56]; *p =* 0.06
Lag1	**0.15 [0.02–0.92];** ***p** **=*** **0.04**	**0.27 [0.08–0.89];** ***p** **=*** **0.032**	0.68 [0.19–2.48]; *p =* 0.564	0.54 [0.09–3.38]; *p =* 0.512
Lag4	0.73 [0.51–1.03]; *p =* 0.072	0.8 [0.63–1]; *p =* 0.05	1.03 [0.81–1.31]; *p =* 0.792	1.17 [0.83–1.65]; *p =* 0.366
Lag7	1.11 [0.98–1.26]; *p =* 0.088	1.07 [0.99–1.17]; *p =* 0.106	0.97 [0.89–1.06]; *p =* 0.494	0.99 [0.87–1.12]; *p =* 0.89
Lag14	0.96 [0.82–1.13]; *p =* 0.624	**0.88 [0.78–0.99];** ***p** **=*** **0.036**	0.9 [0.79–1.03]; *p =* 0.138	**0.79 [0.66–0.95];** ***p** **=*** **0.012**
**Relative humidity** **over 3 days (mean)**	**37 % (p**_**1**_ **)**	**41 % (p**_**5**_ **)**	**82 % (p**_**95**_ **)**	**89 % (p**_**99**_ **)**
Lag0	1.01 [0.76–1.35]; *p =* 0.936	1.02 [0.82–1.27]; *p =* 0.862	1.02 [0.81–1.27]; *p =* 0.872	1.04 [0.74–1.47]; *p =* 0.81
Lag1	1.18 [0.77–1.83]; *p =* 0.45	1.14 [0.82–1.58]; *p =* 0.426	0.91 [0.65–1.27]; *p =* 0.56	0.87 [0.52–1.44]; *p =* 0.584
Lag4	1.03 [0.92–1.14]; *p =* 0.61	1.15 [0.63–1.57]; *p =* 0.826	0.97 [0.89–1.06]; *p =* 0.55	1.04 [0.92–1.18]; *p =* 0.52
Lag7	**0.94 [0.88–0.99];** ***p** **=*** **0.032**	1.01 [0.97–1.05]; *p =* 0.634	1.05 [1–1.1]; *p =* 0.034	**1.12 [1.05–1.19];** ***p*** **<** **0.001**
Lag14	0.91 [0.82–1.01]; *p =* 0.084	1 [0.93–1.08]; *p =* 0.924	0.95 [0.89–1.03]; *p =* 0.206	0.95 [0.85–1.07]; *p =* 0.416
**Precipitation** **over 3 days (sum)**	–	–	**24 mm (p**_**95**_ **)**	**40 mm (p**_**99**_ **)**
Lag0	–	–	0.97 [0.8–1.17]; *p =* 0.732	0.82 [0.57–1.19]; *p =* 0.304
Lag1	–	–	0.9 [0.7–1.14]; *p =* 0.368	0.92 [0.58–1.46]; *p =* 0.726
Lag4	–	–	**0.91 [0.84–0.99];** ***p** **=*** **0.028**	1.06 [0.92–1.23]; *p =* 0.394
Lag7	–	–	0.98 [0.93–1.04]; *p =* 0.506	0.94 [0.85–1.04]; *p =* 0.244
Lag14	–	–	0.95 [0.87–1.05]; *p =* 0.316	0.98 [0.81–1.18]; *p =* 0.808
**Mean wind speed** **over 3 days (mean)**	**1.4 m/s (p**_**1**_ **)**	**1.9 m/s (p**_**5**_ **)**	**5.1 m/s (p**_**95**_ **)**	**6.2 m/s (p**_**99**_ **)**
Lag0	0.95 [0.63–1.44]; *p =* 0.824	1 [0.89–1.11]; *p =* 0.966	0.96 [0.82–1.13]; *p =* 0.632	1.04 [0.78–1.37]; *p =* 0.806
Lag1	0.9 [0.55–1.45]; *p =* 0.65	0.91 [0.79–1.04]; *p =* 0.158	0.99 [0.8–1.23]; *p =* 0.932	1.07 [0.75–1.53]; *p =* 0.704
Lag4	1.02 [0.86–1.2]; *p =* 0.84	0.98 [0.94–1.02]; *p =* 0.402	0.98 [0.92–1.05]; *p =* 0.642	1.02 [0.91–1.14]; *p =* 0.77
Lag7	1.03 [0.92–1.15]; *p =* 0.574	0.98 [0.95–1.01]; *p =* 0.16	0.98 [0.93–1.04]; *p =* 0.506	0.93 [0.85–1.01]; *p =* 0.076
Lag14	**0.71 [0.57–0.88];** ***p** **=*** **0.002**	**0.91 [0.87–0.96]; p < 0.001**	0.97 [0.9–1.04]; *p =* 0.42	1.01 [0.89–1.15]; *p =* 0.856
**Atmospheric pressure over 3 days (mean)**	**980 hPa (p**_**1**_ **)**	**985 hPa (p**_**5**_ **)**	**1008 hPa (p**_**95**_ **)**	**1013 hPa (p**_**99**_ **)**
Lag0	1.18 [0.81–1.71]; *p =* 0.402	1.17 [0.91–1.5]; *p =* 0.23	1.13 [0.84–1.53]; *p =* 0.42	1.05 [0.61–1.81]; *p =* 0.852
Lag1	0.93 [0.49–1.76]; *p =* 0.826	0.89 [0.58–1.38]; *p =* 0.614	0.78 [0.46–1.32]; *p =* 0.354	0.69 [0.28–1.72]; *p =* 0.428
Lag4	0.95 [0.83–1.09]; *p =* 0.494	0.99 [0.9–1.08]; *p =* 0.796	0.93 [0.84–1.03]; *p =* 0.16	0.95 [0.8–1.13]; *p =* 0.59
Lag7	0.97 [0.9–1.03]; *p =* 0.32	**0.95 [0.91–1];** ***p** **=*** **0.03**	1.03 [0.99–1.08]; *p =* 0.186	**1.11 [1.03–1.2];** ***p** **=*** **0.008**
Lag14	0.96 [0.86–1.07]; *p =* 0.474	0.97 [0.9–1.04]; *p =* 0.356	1.03 [0.96–1.1]; *p =* 0.48	0.91 [0.81–1.03]; *p =* 0.146

Percentiles (1st, 5th, 95th and 99th) for extreme weather conditions were calculated by three-day averaging of mean temperature, relative humidity mean wind-speed or atmospheric pressure or three-day sum in case of precipitation. Brackets contain confidence intervals (95%); Significant results (p ≤ 0.05) are highlighted.

In summary, the data highlights that prolonged cold spells of 3 days increase the risk of AOM-related EVs within one day of the temperature event. Prolonged heat waves showed a cumulative increased risk for AOM-related EVs 4 to 7 days after the weather event.

### Relative humidity

Subsequently, we were interested in the effects of extreme relative humidity conditions on AOM-related EVs (Figure 2B). At low humidity of 34% (p_1_) and 39% (p_5_), no significant immediate nor delayed effect was noticed for 14 days after the weather event, with the RR ranging from 1.14 [0.79–1.65] and 1.09 [0.86–1.37] on the same day to 0.94 [0.75–1.17] and 1.04 [0.90–1.20] on day 14, respectively (*p* > 0.05). High relative humidity events of 86% (p_95_) showed no significant change in RR and ranged from 0.88 [0.70–1.11] on the same day to 0.96 [0.84–1.10] 14 days after the event (*p* > 0.05). Extremely high humidity events of 92% (p_99_) showed a significantly increased RR of 1.14 [1.01−1.28; *p* = 0.028] 7 days after the weather event (Table 2). No significant effect on the cRR was noticed for either low-humidity or high-humidity events (*p* > 0.05, Supplementary Table 1).

Prolonged low humidity of 37% (p_1_) over three consecutive days resulted in a minor but significant decrease of RR to 0.94 [0.88–0.99; *p* = 0.032] 7 days after the weather event (Figure 3B). Prolonged high humidity events at 89% (p_99_) showed a significant increase in RR to 1.12 [1.05–1.19; *p* < 0.001] 7 days after the event (Table 3). The cRR after prolonged humidity events followed the same trend, being significantly decreased to 0.60 at lag-day 14 after 37% humidity (p_1_), and significantly increased to 1.43 at 89% (p_99_) humidity on lag-day 7 (*p* = 0.012 and 0.034, Supplementary Table 2).

Taken together, the data suggest that prolonged humidity events have a significant effect on the occurrence of AOM-related EVs. While low humidity decreases the occurrence of AOM-related EVs, high humidity results in an increased rate of EVs 7 days after the weather event.

### Precipitation

We have shown that extreme temperatures and humidity were associated with AOM-related EVs, so we next investigated precipitation as a potential influencing factor (Figure 2C). Same day RR were 0.97 [0.79–1.21] at 10mm (p_95_) and 0.80 [0.47–1.36] at 24 mm (p_99_), respectively. No significant effect on RR or cRR was noted at any lag-day over the 14-day observation period (Table 2 and Supplementary Table 1). Prolonged heavy precipitation of 24 mm (p_95_) resulted in a significant reduction of RR to 0.91 [0.84–0.99; *p* = 0.0284] after four days (Table 3). The cRR for AOM-related EVs following prolonged heavy precipitation was similarly significantly decreased from 0.70 to 0.52 (*p* = 0.01 – 0.008) from days 4 to 14 at 24 mm (p_95_) and 0.31 (*p* = 0.026) on day 14 at 40 mm (p_99_, Figure 3C, Supplementary Table 2).

These data point toward a mitigating effect of prolonged heavy precipitation on AOM-related EVs over 14 days, whereas no immediate effect was noticed after one-day-long events.

### Atmospheric pressure

Next, we investigated the association between AOM-related EVs and atmospheric pressure (Figure 2D). Very low atmospheric pressure resulted in a same-day RR of 1.19 [0.77–1.84] to 1.02 [0.78–1.34] at 976hPa (p_1_) and 983hPa (p_5_), respectively. Very high atmospheric pressure resulted in a same-day RR of 1.06, both at 1009hPa (p_95_) and 1014hPa (p_99_) (*p* > 0.05). No significant change in RR or cRR at extremely low or extremely high atmospheric pressure was noticed at any lag-day over the 14-day observation period (Table 2 and Supplementary Table 1). Prolonged low atmospheric pressure of 985hPa (p_5_) over three days showed a significantly decreased RR to 0.95 [0.91–1.00; *p* = 0.03] seven days after the weather event (Figure 3D). High atmospheric pressure of 1008hPa (p_95_) over three days resulted in a significantly increased RR of 1.11 [1.03–1.20; *p* = 0.008] 7 days after the weather event (Table 3). Prolonged atmospheric pressure events did not significantly alter the cRR at any lag period over 14 days (Supplementary Table 2).

Prolonged atmospheric pressure events therefore showed a significant impact on the RR of AOM-related EVs, decreasing at low pressure events and increasing at high pressure events.

### Mean wind speed

Finally, we investigated the impact of mean wind speed as a potential factor in AOM-related EVs (Figure 2E). Low mean wind speeds of 1.0 m/s (p_1_) and 1.5 m/s (p_5_) from the same day up to day 14 ranged from 0.84 to 0.93 and 0.97 to 0.97, respectively (*p* > 0.05). At wind speeds of 5.9 m/s (p_95_) and 7.5 m/s (p_99_) the RR ranged from 1.06 to 0.95 and 0.75 to 1.22 over the 14-day observation period (*p* > 0.05, Table 2). cRR similarly showed no significant change over the 14-day lag period (Supplementary Table 1). Prolonged low wind speeds over three days showed a significant decrease in RR to 0.71 [0.57–0.88; *p* = 0.002] at 1.4 m/s (p_1_) and 0.91 [0.87–0.96; *p* < 0.001] at 1.9 m/s (p_5_), 14 days after the weather event (Figure 3E, Table 3). High average wind speeds of 5.1 m/s (p_95_) over three days showed a significant decrease in cRR to 0.51 [0.32–0.79; *p* = 0.002] within 14 days after the event (Supplementary Table 2).

Taken together, mean wind speeds did not significantly affect the risk for AOM-related EVs after a single-day event. Prolonged low wind speeds and high wind speeds, however, were associated with a lower risk of EVs within 14 days after the weather event.

### Seasonal differences

Overall, the data, stratified by summer and winter months, followed a similar pattern to the whole-year data. In contrast to the annual model, in the summer months, events with high temperatures significantly increased the occurrence of AOM-related EVs 4 days after the event. Relative humidity followed the same trend in both summer and winter, with low humidity events in summer significantly decreasing the RR for AOM-related EVs after both 1-day and 3-day events. In contrast, high humidity in winter significantly increased the occurrence of AOM-related EVs 7 days after the event. Heavy precipitation over 3 days, both in summer and winter, significantly decreased the RR for AOM-related EVS for up to 14 days after the weather event. Concerning wind speeds, AOM-related EVs in summer and winter showed a similar pattern in RR compared to year-round results. In addition, low-pressure events significantly reduced AOM-related EVs in both summer and winter, similar to the year-round data. For a detailed overview, see Supplementary Tables 3–6.

## Discussion

In this study, we investigated immediate and delayed effects of extreme weather conditions on AOM-related EVs in Vienna, Austria. Austria features a temperate continental climate with cold winters and warm summers. AOM-related EVs were occurring in higher frequency in winter and spring (Dec-May) compared to summer and autumn (June – Nov). In line with our results, the seasonality of AOM occurrence is well documented in children and adults (5, 8, 10, 18). Moreover, web-based search queries for middle ear infection and possible treatment options have shown a similar seasonality with a peak-interest in winter months (30).

Despite its seasonality, the relationship of AOM-related EVs to meteorological variables needs to be clarified. Overall, mean temperature and relative humidity followed a clear seasonal character, featuring high temperatures and low humidity in summer months, and vice versa in winter months. Precipitation, atmospheric pressure, and mean wind speed were more heterogenous with considerable variation between the years. In previous studies, the occurrence of AOM-related EVs showed a weak, but significant, negative association with temperature and relative humidity (8, 17, 18). However, these studies either correlated mean monthly weather variables to AOM-related EVs, thus ignoring daily weather variations within each month, or focused their investigation on AOM-related EVs in children and adolescents. Limiting the study cohort, however, neglects the 20–30% of adult patients with AOM-related symptoms who visit the emergency department, presenting a significant burden on healthcare (6, 10, 31). Additionally, of all patients presenting with complications due to AOM, 76% have been shown to be adults, wo might require potentially invasive surgical management for disease control (10). In this study, we, therefore, evaluated the risk for AOM-related EVs following extreme weather events in a heterogeneous patient cohort over 14 days. We applied a DLNM model to account for the complexity of weather on disease incidence and assessed its effects after single-day and prolonged events over three days. By stratifying our data into summer and winter months, we further elucidated on possible seasonal differences in the impact of extreme weather events on AOM-related EVs. Interestingly, for most weather events, the RR of AOM-related EVs followed a similar pattern to the annual data.

We provided evidence that one-day events of extremely high or low temperatures did not significantly affect the occurrence of AOM-related EVs. However, following prolonged cold spells of three days, very low and low mean temperatures significantly and markedly increased the RR of AOM-related EVs on the same day, suggesting that AOM symptoms require prolonged low temperature conditions to develop. These results add to the previous body of literature, describing an increased risk of AOM-related EVs with low temperatures. Tian et al. showed a correlation between low temperatures and AOM-related EVs in a large cohort of over 10000 patients in Lanzhou, China (18). Similarly, Gestro et al. (8) found a significant correlation between low temperatures and AOM-related EVs over three days in children and adolescents in Cuneo, Italy (8).

The mechanism of cold temperatures increasing AOM occurrence likely stems from its relation to URIs, which follow seasonal patterns as well (32). Viral pathogens that cause URI play a critical role in the etiology of AOM and are regularly detected in middle ear fluid during infection (13). The negative correlation between temperature and common viruses, such as the RSV, or the common cold has previously been described in the literature (33). Cold air cools the nasal mucosa and has been shown to compromise mucociliary clearance, one of the leading defense mechanisms of the upper respiratory system (34) which results in decreased evacuation of pathogens from the nasal mucosa and facilitates possible invasion and infection. The activity of phagocytes and leucocytes, presenting the non-specific local immune response to pathogens, is similarly decreased by low temperatures (35, 36). After infection with RSV and influenza viruses, a striking occurrence of ETD was observed in healthy subjects, promoting a significant risk factor for developing AOM (14, 37). Furthermore, the temporal development of AOM has been noticed 3–4 days after URI (12). The significantly increased RR for AOM-related EVs only after a three-day cold spell may, therefore, be indicative of the required duration for the development of AOM after URI.

Interestingly, seven days after prolonged heat waves of 30°C, we also observed a minor but significant increase in AOM-related EVs. In the presented study, based on the patient's digital medical charts during EVs, we could not distinguish between acute otitis media and chronic suppurative otitis media exacerbation. Chronic suppurative otitis media presents similar symptoms and results from middle-ear infection through a persistent perforation of the eardrum of residing bacteria of the outer ear canal or surrounding environment. Warmer temperatures and increased humidity are both associated with an increase in infections originating from the outer ear canal over seven days following the weather event, which might explain our significant finding (38). This finding was reconfirmed in summer months after stratification into the seasonal models, with an increased RR 4 days after high temperature events.

Similar to the data regarding temperature, one-day low humidity events had no impact on AOM-related EVs, while prolonged low humidity over three days significantly decreased EVs one week after. Also, a significantly higher EV rate after prolonged high humidity events was observed, suggesting a positive correlation between humidity and AOM-related EVs. In a similar climate to Vienna, in Cuneo, Italy, no correlation was found between daily relative humidity and AOM-related EVs (8). In contrast to our results, Jiang et al. found an inverse correlation between relative humidity and AOM-related EVs in Shanghai, which, unlike Austria, features an annual high relative humidity between 78 and 84% (17). In Austria's continental climate, low humidity events occur in summer at high temperatures and the lowest annual rate AOM-related EVs. Prolonged high-humidity events, therefore, occur in winter and may not be causal for the increase in AOM-related events. Indoor heating reduces relative indoor humidity, which has been shown to increase the risk for AOM during the heating season in pediatric patients (39). Therefore, the high relative humidity may not directly affect the occurrence of AOM, but may be a reflection of cold temperatures and the heating season in the winter months, increasing the risk for AOM.

In the case of precipitation, AOM-related EVs on the same day showed a non-significant trend toward lower EVs. At the same time a significant decrease was noticed four days after continuous heavy rainfalls over three days. In Vienna, snow accounts for a significant portion of precipitation during winter. Based on the available weather data, we could not differentiate between snowfall and rain. The effect of precipitation in the form of rain may, therefore, be underrepresented in our study. While the effect of heavy precipitation on AOM-related EVs has not yet been described, previous studies showed a mild but significant reduction in the overall risk of EVs in children and adults after heavy rain and snowstorms (40, 41). Precipitation may, therefore, directly influence the incidence of AOM-related EVs or could be a consequence of human behavioral patterns, as people may be more reluctant to seek medical attention during rain or snow.

The effects of atmospheric pressure and wind speed on AOM-related EVs have, so far, been sparsely investigated. Similar to previous results, low atmospheric pressure in our study significantly decreased AOM-related EVs 7 days after prolonged events. In contrast, high pressure increased the rate of AOM-related EVs during the same period (18). The middle ear space is a tightly regulated pressure system. Under physiological conditions, gas diffusion across the mucosa allows for a steady pressure state between the middle ear and atmospheric pressure. Rapid changes in pressure are compensated by the reflexive opening of the eustachian tube (42). It is possible that ETD, caused by mucosal swelling during URI, causes a failure in pressure-equalization during pressure changes, resulting in pain and AOM-like symptoms. Consistent with this reasoning, infectious diseases such as URI and asthma exacerbation are similarly positively correlated to atmospheric pressure changes (43, 44). A different possibility for this significant finding could be the interaction between atmospheric pressure and air temperature, which are tightly correlated. During hot temperatures, air density is reduced, which results in a decrease in local atmospheric pressure. Similarly, at cold temperatures, an increase in atmospheric pressure is noted as the air density accumulates. It is, therefore, unclear whether atmospheric pressure is correlative or causative for the occurrence of AOM-related EVs, which warrants further investigation.

Regarding wind speed, our data does not show any significant effect of very low or high mean wind speeds on AOM-related EVs for single-day events. Similarly, Tian et al. and Gestro et al. did not find a significant correlation with wind speed after single-day events (8, 18). Prolonged low wind speeds, however, were associated with a moderately reduced risk of EVs, being significant 14 days after the weather event. Prolonged periods of low wind speeds might reduce the dispersion of particular matter, which is a significant risk factor for URI and AOM (45, 46). Calm wind conditions could reduce inhaled irritants, thereby reducing upper respiratory tract inflammation. In line with this argument, Wenfang et al. found a strong correlation between wind-speed and URI, suggesting an increased infection rate during high winds (44).

Our study has several limitations. Firstly, despite featuring a heterogenous patient cohort, the single-center and retrospective study design poses a limiting factor on translation into other study populations. Our results stem from a tertiary care center with a separate pediatric emergency care unit. This might pose a selection bias toward adult patients and more severe cases of AOM, explaining the relatively high occurrence of mastoiditis in our patient cohort. Also, it must be acknowledged that this study examined the overall relative risk for AOM-related EVs of an entire population, so interpretation of the individual risk is not possible. Next, we used the data from a single weather station to represent the weather effects in our population, which could potentially introduce exposure misclassification on the individual level due to spatial weather differences across the entire Viennese urban area. Also, Vienna features a dry continental climate with cold winters. Care must be taken when translating our results into regions with different climates. Additionally, this study only examined the effects of extreme weather events, making the results not applicable to the effects of moderate weather conditions. In addition, other time-varying variables such as air pollution were not considered in the study. Previously, in urban areas with heavy air pollution, a clear correlation between particulate matter, gaseous pollutants, and the occurrence of AOM could be established (18). In less polluted areas however, only negligible effects of NO_2_, O_3_ and PM_10_ could be observed (8). Given the low emission rates in Vienna compared to other large cities, we suspect that these confounders would therefore only insignificantly affect the measured outcomes, even though they cannot be disregarded (47). Finally, the DLNM used in this study is designed for time series data of single variables. As such, our study investigated the effects of each individual weather variable on AOM-related EVs along the time domain, which could possibly omit combined effects of different weather variables.

Taken together, to the best of our knowledge, this is the first study to investigate the effects of single-day and prolonged extreme weather events on AOM-related EVs. While only a minor impact of one-day long weather events on the rate of AOM-related EVs was noticed by relative humidity, we discovered that prolonged mean temperature, humidity precipitation, wind speed and atmospheric pressure all significantly influence the occurrence of AOM-related EVs. Additionally, a same-day effect was observed only after prolonged low temperatures, underscoring the importance of delayed effects of extreme weather events on AOM.

## Conclusions

Single-day extreme weather events had no significant effects on AOM-related EVs. On the other hand, prolonged extremely low temperatures, high relative humidity and high atmospheric pressure increased the risk for EVs. Low relative humidity, heavy precipitation, low wind-speed and low atmospheric pressure events further diminished EV risk. A same-day effect was only noticed for prolonged extremely low temperatures, while the impact of the other meteorological conditions was observed over 14 subsequent days. These results could aid in optimally allocating resources in healthcare management of similar climates and contribute to educating patients on the underlying environmental factors responsible for AOM. Our results warrant further investigation into the immediate and delayed meteorological effects on acute otitis media within a larger study population and different climates.

## Data availability statement

The raw data supporting the conclusions of this article will be made available by the authors, without undue reservation.

## Author contributions

MN: concept of study, analysis of results, write up of manuscript, and critical review of all contents. ML and FP: concept of study, collection of data, and critical review of all contents. MH, FB, TP, and DR: concept of study, analysis of results, and critical review of all contents. DL: concept of study, collection of data, analysis of results, write up of manuscript, and critical review of all contents. All authors contributed to the article and approved the submitted version.
