# Supplementary material for: Fluctuations in emergency department visits related to acute otitis media are associated with extreme meteorological conditions
Source: Front Public Health. 2023 Jun 1;11:1153111. doi: 10.3389/fpubh.2023.1153111 (PMC10267338; doi:10.3389/fpubh.2023.1153111)
Supplement: Supplementary file 1 [file Table_1.docx]

***Supplementary Tables***

**Fluctuations in emergency department visits related to Acute otitis media are associated with extreme meteorological conditions**

Michael Nieratschker, MD^1^, Markus Haas, BA^1^, Mateo Lucic^1^, Franziska Pichler^1^, Faris F. Brkic, MD PhD^1^, Thomas Parzefall, MD PhD^1^, Dominik Riss, MD^1*^, David T. Liu, MD PhD^1^

1. Department of Otorhinolaryngology, Head and Neck Surgery, Medical University of Vienna, Vienna, Austria

| **Cumulative Relative risk (cRR) of AOM-related EVs** | | | | |
| --- | --- | --- | --- | --- |
| **Mean temperature** | **-5 °C (p_1_)** | **0 °C (p_5_)** | **27 °C (p_95_)** | **30 °C (p_99_)** |
| Lag0 | 1.13 [0.49-2.61]; p=0.78 | 1.15 [0.7-1.9]; p=0.58 | 1.33 [0.77-2.3]; p=0.306 | 1.52 [0.74-3.11]; p=0.252 |
| Lag0-1 | 0.88 [0.39-2]; p=0.758 | 0.78 [0.46-1.32]; p=0.356 | 1.23 [0.72-2.09]; p=0.454 | 1.35 [0.66-2.75]; p=0.406 |
| Lag0-4 | 0.76 [0.39-1.49]; p=0.424 | 0.88 [0.56-1.37]; p=0.564 | 1.15 [0.72-1.85]; p=0.556 | 1.74 [0.94-3.19]; p=0.076 |
| Lag0-7 | 0.87 [0.44-1.73]; p=0.694 | 0.85 [0.53-1.39]; p=0.524 | 1.03 [0.61-1.73]; p=0.912 | 1.68 [0.85-3.32]; p=0.132 |
| Lag0-14 | 0.89 [0.38-2.12]; p=0.802 | 0.79 [0.42-1.49]; p=0.46 | 1.07 [0.52-2.2]; p=0.854 | 1.46 [0.53-4.05]; p=0.462 |
| **Relative humidity** | **34 % (p_1_)** | **39 % (p_5_)** | **86 % (p_95_)** | **92 % (p_99_)** |
| Lag0 | 1.14 [0.79-1.65]; p=0.484 | 1.09 [0.86-1.37]; p=0.488 | 0.88 [0.7-1.11]; p=0.28 | 1.03 [0.71-1.5]; p=0.878 |
| Lag0-1 | 1.13 [0.74-1.74]; p=0.572 | 1.09 [0.82-1.43]; p=0.556 | 1.02 [0.77-1.36]; p=0.874 | 1.02 [0.64-1.62]; p=0.94 |
| Lag0-4 | 0.99 [0.59-1.65]; p=0.972 | 0.96 [0.66-1.39]; p=0.826 | 0.76 [0.53-1.11]; p=0.154 | 0.9 [0.51-1.6]; p=0.724 |
| Lag0-7 | 0.67 [0.36-1.24]; p=0.2 | 0.99 [0.63-1.57]; p=0.974 | 0.89 [0.58-1.39]; p=0.616 | 1.48 [0.72-3.02]; p=0.286 |
| Lag0-14 | 0.5 [0.21-1.19]; p=0.118 | 0.96 [0.49-1.89]; p=0.904 | 0.88 [0.46-1.68]; p=0.698 | 1.06 [0.38-2.98]; p=0.91 |
| **Precipitation** | - | - | **10 mm (p_95_)** | **24 mm (p_99_)** |
| Lag0 | - | - | 0.97 [0.79-1.21]; p=0.804 | 0.8 [0.47-1.36]; p=0.408 |
| Lag0-1 | - | - | 0.95 [0.71-1.27]; p=0.738 | 0.63 [0.3-1.35]; p=0.234 |
| Lag0-4 | - | - | 0.83 [0.54-1.28]; p=0.41 | 0.43 [0.12-1.51]; p=0.186 |
| Lag0-7 | - | - | 0.61 [0.36-1.06]; p=0.078 | 0.64 [0.13-3.08]; p=0.576 |
| Lag0-14 | - | - | 0.5 [0.22-1.14]; p=0.1 | 0.67 [0.07-6.86]; p=0.736 |
| **Mean wind speed** | **1.0 m/s (p_1_)** | **1.5 m/s (p_5_)** | **5.9 m/s (p_95_)** | **7.5 m/s (p_99_)** |
| Lag0 | 0.84 [0.64-1.11]; p=0.218 | 0.97 [0.89-1.06]; p=0.536 | 1.06 [0.84-1.33]; p=0.638 | 0.75 [0.39-1.45]; p=0.396 |
| Lag0-1 | 0.76 [0.52-1.12]; p=0.17 | 0.91 [0.81-1.02]; p=0.12 | 0.89 [0.65-1.23]; p=0.478 | 0.88 [0.36-2.17]; p=0.786 |
| Lag0-4 | 0.78 [0.42-1.44]; p=0.424 | 0.92 [0.77-1.11]; p=0.4 | 1 [0.61-1.63]; p=0.992 | 0.78 [0.2-3.04]; p=0.716 |
| Lag0-7 | 0.7 [0.31-1.57]; p=0.382 | 0.9 [0.7-1.15]; p=0.396 | 0.96 [0.5-1.85]; p=0.914 | 0.45 [0.08-2.63]; p=0.378 |
| Lag0-14 | 0.36 [0.11-1.26]; p=0.11 | 0.75 [0.51-1.1]; p=0.138 | 0.81 [0.3-2.2]; p=0.676 | 0.54 [0.04-7.09]; p=0.636 |
| **Atmospheric pressure** | **976 hPa (p_1_)** | **983 hPa (p_5_)** | **1009 hPa (p_95_)** | **1014 hPa (p_99_)** |
| Lag0 | 1.19 [0.77-1.84]; p=0.446 | 1.02 [0.78-1.34]; p=0.868 | 1.06 [0.8-1.41]; p=0.674 | 1.06 [0.62-1.8]; p=0.836 |
| Lag0-1 | 1.19 [0.72-1.96]; p=0.506 | 0.93 [0.69-1.26]; p=0.638 | 0.83 [0.61-1.12]; p=0.226 | 0.67 [0.39-1.15]; p=0.152 |
| Lag0-4 | 1.3 [0.63-2.68]; p=0.472 | 0.84 [0.57-1.24]; p=0.374 | 0.76 [0.55-1.06]; p=0.106 | 0.68 [0.38-1.23]; p=0.202 |
| Lag0-7 | 0.98 [0.38-2.52]; p=0.96 | 0.76 [0.47-1.25]; p=0.284 | 0.83 [0.55-1.24]; p=0.36 | 0.79 [0.37-1.68]; p=0.542 |
| Lag0-14 | 1.06 [0.26-4.32]; p=0.932 | 0.69 [0.33-1.44]; p=0.324 | 0.89 [0.48-1.63]; p=0.698 | 0.67 [0.21-2.13]; p=0.498 |

**Suppl. Table 1.** Cumulative relative risk for acute otitis media-related EVs under extreme weather conditions (1st, 5th, 95th and 99th percentile) compared to median conditions. Brackets contain confidence intervals (95%)

| **Cumulative Relative risk (cRR) of AOM-related EVs**  **after prolonged extreme weather conditions over 3 days** | | | | |
| --- | --- | --- | --- | --- |
| **Mean temperature**  **over 3 days (mean)** | **-4 °C (p_1_)** | **0 °C (p_5_)** | **26 °C (p_95_)** | **30 °C (p_99_)** |
| Lag0 | **3.15 [1.26-7.88]; p=0.014** | **2.14 [1.14-4.04]; p=0.018** | 2 [1-4]; p=0.052 | 2.51 [0.96-6.56]; p=0.06 |
| Lag0-1 | 0.47 [0.16-1.37]; p=0.17 | 0.57 [0.28-1.16]; p=0.12 | 1.37 [0.66-2.85]; p=0.406 | 1.36 [0.48-3.9]; p=0.564 |
| Lag0-4 | 0.86 [0.59-1.26]; p=0.434 | 0.96 [0.72-1.27]; p=0.754 | 1.33 [0.98-1.82]; p=0.068 | **2 [1.32-3.04]; p=0.002** |
| Lag0-7 | 1.05 [0.74-1.5]; p=0.778 | 1.01 [0.77-1.34]; p=0.928 | 1.03 [0.76-1.4]; p=0.838 | **1.69 [1.11-2.57]; p=0.014** |
| Lag0-14 | 1.06 [0.7-1.62]; p=0.778 | 0.91 [0.64-1.29]; p=0.59 | 1.26 [0.84-1.88]; p=0.266 | 1.62 [0.9-2.92]; p=0.11 |
| **Relative humidity**  **over 3 days (mean)** | **37 % (p_1_)** | **41 % (p_5_)** | **82 % (p_95_)** | **89 % (p_99_)** |
| Lag0 | 1.01 [0.76-1.35]; p=0.936 | 1.02 [0.82-1.27]; p=0.862 | 1.02 [0.81-1.27]; p=0.872 | 1.04 [0.74-1.47]; p=0.81 |
| Lag0-1 | 1.2 [0.89-1.61]; p=0.236 | 1.16 [0.93-1.45]; p=0.178 | 0.92 [0.74-1.16]; p=0.48 | 0.9 [0.64-1.29]; p=0.578 |
| Lag0-4 | 1.03 [0.81-1.3]; p=0.812 | 1.16 [0.96-1.4]; p=0.114 | 0.84 [0.69-1.02]; p=0.076 | 0.89 [0.67-1.19]; p=0.432 |
| Lag0-7 | 0.82 [0.62-1.08]; p=0.154 | 1.15 [0.63-1.57]; p=0.826 | 0.94 [0.75-1.17]; p=0.556 | **1.43 [1.03-2]; p=0.034** |
| Lag0-14 | **0.6 [0.4-0.89]; p=0.012** | 1.31 [0.96-1.79]; p=0.094 | 0.97 [0.71-1.31]; p=0.836 | 1.07 [0.67-1.71]; p=0.772 |
| **Precipitation**  **over 3 days (sum)** | - | - | **24 mm (p_95_)** | **40 mm (p_99_)** |
| Lag0 | - | - | 0.97 [0.8-1.17]; p=0.732 | 0.82 [0.57-1.19]; p=0.304 |
| Lag0-1 | - | - | 0.87 [0.69-1.08]; p=0.2 | 0.76 [0.5-1.15]; p=0.196 |
| Lag0-4 | - | - | **0.7 [0.54-0.92]; p=0.01** | 0.68 [0.4-1.15]; p=0.148 |
| Lag0-7 | - | - | **0.63 [0.45-0.89]; p=0.008** | 0.69 [0.36-1.34]; p=0.27 |
| Lag0-14 | - | - | **0.52 [0.31-0.86]; p=0.012** | **0.31 [0.11-0.87]; p=0.026** |
| **Mean wind speed**  **over 3 days (mean)** | **1.4 m/s (p_1_)** | **1.9 m/s (p_5_)** | **5.1 m/s (p_95_)** | **6.2 m/s (p_99_)** |
| Lag0 | 0.95 [0.63-1.44]; p=0.824 | 1 [0.89-1.11]; p=0.966 | 0.96 [0.82-1.13]; p=0.632 | 1.04 [0.78-1.37]; p=0.806 |
| Lag0-1 | 0.85 [0.55-1.32]; p=0.48 | 0.9 [0.8-1.01]; p=0.082 | 0.95 [0.8-1.13]; p=0.578 | 1.11 [0.82-1.51]; p=0.508 |
| Lag0-4 | 1 [0.58-1.73]; p=0.996 | 0.91 [0.79-1.03]; p=0.146 | 0.9 [0.72-1.11]; p=0.314 | 1.23 [0.83-1.84]; p=0.306 |
| Lag0-7 | 1.05 [0.53-2.09]; p=0.894 | 0.86 [0.72-1.03]; p=0.098 | 0.8 [0.6-1.06]; p=0.12 | 1.03 [0.61-1.73]; p=0.91 |
| Lag0-14 | 0.45 [0.16-1.3]; p=0.142 | **0.57 [0.44-0.75]; p<0.001** | **0.51 [0.32-0.79]; p=0.002** | 0.78 [0.36-1.69]; p=0.526 |
| **Atmospheric pressure**  **over 3 days (mean)** | **980 hPa (p_1_)** | **985 hPa (p_5_)** | **1008 hPa (p_95_)** | **1013 hPa (p_99_)** |
| Lag0 | 1.18 [0.81-1.71]; p=0.402 | 1.17 [0.91-1.5]; p=0.23 | 1.13 [0.84-1.53]; p=0.42 | 1.05 [0.61-1.81]; p=0.852 |
| Lag0-1 | 1.09 [0.74-1.61]; p=0.648 | 1.04 [0.8-1.35]; p=0.752 | 0.88 [0.65-1.2]; p=0.43 | 0.73 [0.43-1.24]; p=0.246 |
| Lag0-4 | 1.19 [0.89-1.59]; p=0.24 | 1.01 [0.84-1.21]; p=0.946 | 0.85 [0.7-1.02]; p=0.072 | 0.75 [0.53-1.05]; p=0.09 |
| Lag0-7 | 0.99 [0.69-1.42]; p=0.968 | 0.84 [0.67-1.05]; p=0.118 | 0.92 [0.74-1.15]; p=0.474 | 0.99 [0.65-1.51]; p=0.966 |
| Lag0-14 | 0.96 [0.57-1.63]; p=0.884 | 0.76 [0.55-1.05]; p=0.092 | 0.97 [0.7-1.33]; p=0.832 | 0.95 [0.51-1.78]; p=0.872 |

**Suppl. Table 2.** Cumulative relative risk for acute otitis media-related EVs after prolonged extreme weather conditions compared to median conditions. Percentiles (1st, 5th, 95th and 99th) for extreme weather conditions were calculated by three-day averaging of mean temperature, relative humidity mean wind-speed or atmospheric pressure or three-day sum in case of precipitation. Brackets contain confidence intervals (95%); Significant results (p≤0.05) are highlighted

| **Relative risk (RR) of AOM-related EVs in Summer months** | | | | |
| --- | --- | --- | --- | --- |
| **Mean temperature** | **7°C (p_1_)** | **11 °C (p_5_)** | **28 °C (p_95_)** | **30 °C (p_99_)** |
| Lag0 | 1.19 [0.43-3.3]; p=0.742 | 0.65 [0.33-1.27]; p=0.204 | 1.28 [0.77-2.12]; p=0.348 | 1.39 [0.64-3.02]; p=0.406 |
| Lag1 | 1.42 [0.34-6.01]; p=0.634 | 1.77 [0.78-4]; p=0.172 | 1.03 [0.51-2.07]; p=0.936 | 0.8 [0.28-2.29]; p=0.676 |
| Lag4 | 0.99 [0.65-1.51]; p=0.964 | 1.13 [0.87-1.46]; p=0.362 | 1.17 [0.96-1.43]; p=0.118 | **1.38 [1.02-1.86]; p=0.036** |
| Lag7 | 0.97 [0.73-1.29]; p=0.832 | 1.05 [0.9-1.22]; p=0.52 | 1.03 [0.91-1.16]; p=0.664 | 1.02 [0.85-1.22]; p=0.812 |
| Lag14 | 1.01 [0.7-1.46]; p=0.962 | 1.02 [0.78-1.33]; p=0.908 | 0.86 [0.69-1.08]; p=0.202 | 0.89 [0.61-1.29]; p=0.526 |
| **Relative humidity** | **32 % (p_1_)** | **37 % (p_5_)** | **75 % (p_95_)** | **83 % (p_99_)** |
| Lag0 | 1.34 [0.78-2.33]; p=0.292 | 1.2 [0.88-1.65]; p=0.256 | 0.82 [0.57-1.19]; p=0.302 | 0.93 [0.6-1.45]; p=0.756 |
| Lag1 | 1 [0.53-1.86]; p=0.988 | 0.9 [0.63-1.28]; p=0.548 | 1.12 [0.79-1.57]; p=0.526 | 0.93 [0.54-1.59]; p=0.786 |
| Lag4 | 0.97 [0.77-1.23]; p=0.814 | 0.93 [0.8-1.08]; p=0.312 | 0.96 [0.78-1.19]; p=0.73 | 1.02 [0.79-1.31]; p=0.908 |
| Lag7 | **0.82 [0.67-1]; p=0.048** | 0.95 [0.85-1.06]; p=0.366 | 0.96 [0.81-1.14]; p=0.63 | 0.95 [0.81-1.12]; p=0.546 |
| Lag14 | 0.87 [0.62-1.22]; p=0.418 | 0.98 [0.8-1.19]; p=0.816 | 1.18 [0.91-1.51]; p=0.21 | 1.07 [0.81-1.41]; p=0.642 |
| **Precipitation** | - | - | **13 mm (p_95_)** | **30 mm (p_99_)** |
| Lag0 | - | - | 0.92 [0.65-1.29]; p=0.626 | 1.13 [0.3-4.27]; p=0.856 |
| Lag1 | - | - | 0.87 [0.62-1.24]; p=0.448 | 0.76 [0.33-1.77]; p=0.532 |
| Lag4 | - | - | 0.91 [0.75-1.1]; p=0.324 | 0.69 [0.37-1.29]; p=0.246 |
| Lag7 | - | - | 0.87 [0.74-1.01]; p=0.07 | 0.85 [0.51-1.41]; p=0.528 |
| Lag14 | - | - | 0.99 [0.78-1.26]; p=0.952 | 0.57 [0.21-1.53]; p=0.264 |
| **Mean wind speed** | **1.0 m/s (p_1_)** | **1.5 m/s (p_5_)** | **5.2 m/s (p_95_)** | **6.7 m/s (p_99_)** |
| Lag0 | 1.2 [0.76-1.89]; p=0.43 | 1.02 [0.76-1.37]; p=0.9 | 0.8 [0.57-1.12]; p=0.196 | 1.03 [0.57-1.83]; p=0.932 |
| Lag1 | 0.91 [0.54-1.5]; p=0.7 | 0.97 [0.72-1.33]; p=0.872 | 0.85 [0.6-1.21]; p=0.366 | 0.74 [0.36-1.53]; p=0.414 |
| Lag4 | 0.93 [0.67-1.3]; p=0.688 | 1.03 [0.86-1.25]; p=0.722 | 1.06 [0.86-1.31]; p=0.596 | 0.81 [0.57-1.15]; p=0.24 |
| Lag7 | 0.93 [0.71-1.22]; p=0.602 | 0.93 [0.8-1.08]; p=0.358 | 0.93 [0.79-1.11]; p=0.44 | 0.78 [0.59-1.03]; p=0.084 |
| Lag14 | 1.13 [0.78-1.62]; p=0.522 | 1.1 [0.88-1.37]; p=0.414 | 1.03 [0.8-1.32]; p=0.82 | 0.82 [0.57-1.2]; p=0.306 |
| **Atmospheric pressure** | **982 hPa (p_1_)** | **986 hPa (p_5_)** | **1004 hPa (p_95_)** | **1008 hPa (p_99_)** |
| Lag0 | 1.23 [0.66-2.3]; p=0.518 | 1.18 [0.81-1.72]; p=0.388 | 1.23 [0.8-1.88]; p=0.346 | 1.43 [0.7-2.94]; p=0.326 |
| Lag1 | 0.51 [0.24-1.08]; p=0.078 | 0.68 [0.44-1.05]; p=0.084 | 0.88 [0.52-1.49]; p=0.626 | 0.7 [0.26-1.88]; p=0.484 |
| Lag4 | **0.69 [0.51-0.93]; p=0.016** | 0.86 [0.72-1.02]; p=0.092 | 0.93 [0.74-1.15]; p=0.494 | **0.55 [0.33-0.93]; p=0.026** |
| Lag7 | 0.87 [0.71-1.06]; p=0.158 | **0.88 [0.78-1]; p=0.046** | 0.99 [0.84-1.16]; p=0.898 | **0.6 [0.39-0.92]; p=0.018** |
| Lag14 | 0.82 [0.56-1.21]; p=0.314 | 1.08 [0.87-1.34]; p=0.47 | 0.98 [0.76-1.25]; p=0.852 | 0.9 [0.57-1.42]; p=0.664 |

**Suppl. Table 3.** Relative risk for acute otitis media-related EVs under extreme weather conditions (1st, 5th, 95th and 99th percentile) in the months from 1^st^ of April until 30^th^ of September compared to median conditions. Brackets contain confidence intervals (95%); Significant results (p≤0.05) are highlighted

| **Relative risk (RR) of AOM-related EVs in Summer months**  **after prolonged extreme weather conditions over 3 days** | | | | |
| --- | --- | --- | --- | --- |
| **Mean temperature**  **over 3 days (mean)** | **7 °C (p_1_)** | **12 °C (p_5_)** | **28 °C (p_95_)** | **30 °C (p_99_)** |
| Lag0 | 0.81 [0.26-2.57]; p=0.722 | 0.5 [0.25-1]; p=0.05 | 1.45 [0.9-2.35]; p=0.13 | 1.59 [0.77-3.3]; p=0.212 |
| Lag1 | 1.89 [0.23-15.31]; p=0.55 | 1.37 [0.42-4.52]; p=0.602 | 0.86 [0.35-2.13]; p=0.748 | 0.68 [0.18-2.66]; p=0.582 |
| Lag4 | 1.09 [0.74-1.6]; p=0.67 | 1.04 [0.83-1.31]; p=0.706 | 1.14 [0.96-1.36]; p=0.14 | 1.22 [0.93-1.61]; p=0.15 |
| Lag7 | 0.97 [0.82-1.16]; p=0.768 | 1.04 [0.95-1.14]; p=0.378 | 1.05 [0.98-1.12]; p=0.172 | 1.1 [0.99-1.23]; p=0.082 |
| Lag14 | 0.94 [0.77-1.15]; p=0.576 | 1.02 [0.89-1.17]; p=0.8 | 0.92 [0.83-1.02]; p=0.122 | 0.86 [0.72-1.03]; p=0.106 |
| **Relative humidity**  **over 3 days (mean)** | **35 % (p_1_)** | **40 % (p_5_)** | **69 % (p_95_)** | **77 % (p_99_)** |
| Lag0 | 0.94 [0.69-1.29]; p=0.704 | 0.92 [0.74-1.13]; p=0.402 | 0.85 [0.66-1.09]; p=0.208 | 0.86 [0.59-1.28]; p=0.466 |
| Lag1 | 1.21 [0.78-1.9]; p=0.396 | 1.06 [0.8-1.42]; p=0.666 | 1.07 [0.75-1.53]; p=0.712 | 1.2 [0.67-2.16]; p=0.538 |
| Lag4 | 0.94 [0.83-1.07]; p=0.358 | 0.98 [0.9-1.06]; p=0.592 | 0.98 [0.89-1.08]; p=0.65 | 0.93 [0.8-1.08]; p=0.324 |
| Lag7 | **0.89 [0.83-0.96]; p=0.002** | **0.95 [0.9-1]; p=0.048** | 1 [0.94-1.06]; p=0.968 | 1.04 [0.96-1.12]; p=0.326 |
| Lag14 | 0.9 [0.78-1.03]; p=0.116 | 1.02 [0.94-1.11]; p=0.634 | 1.02 [0.92-1.12]; p=0.756 | 1.04 [0.92-1.18]; p=0.498 |
| **Precipitation**  **over 3 days (sum)** | - | - | **29 mm (p_95_)** | **46 mm (p_99_)** |
| Lag0 | - | - | 0.99 [0.78-1.25]; p=0.938 | 0.66 [0.36-1.23]; p=0.19 |
| Lag1 | - | - | 1.02 [0.76-1.37]; p=0.904 | 0.63 [0.28-1.4]; p=0.258 |
| Lag4 | - | - | **0.86 [0.77-0.97]; p=0.012** | 0.94 [0.68-1.32]; p=0.736 |
| Lag7 | - | - | 0.95 [0.88-1.03]; p=0.202 | 1.04 [0.86-1.27]; p=0.676 |
| Lag14 | - | - | 1 [0.89-1.13]; p=0.942 | 0.72 [0.47-1.1]; p=0.126 |
| **Mean wind speed**  **over 3 days (mean)** | **1.5 m/s (p_1_)** | **1.9 m/s (p_5_)** | **4.8 m/s (p_95_)** | **5.6 m/s (p_99_)** |
| Lag0 | 1.27 [0.9-1.78]; p=0.176 | 1.04 [0.84-1.28]; p=0.722 | 1.09 [0.86-1.38]; p=0.49 | 1.45 [0.96-2.2]; p=0.076 |
| Lag1 | 1.28 [0.84-1.95]; p=0.252 | 1.07 [0.82-1.39]; p=0.628 | 0.95 [0.7-1.3]; p=0.76 | 0.96 [0.55-1.66]; p=0.874 |
| Lag4 | 1 [0.85-1.18]; p=0.982 | 1.01 [0.93-1.1]; p=0.848 | 1.02 [0.91-1.13]; p=0.764 | 0.93 [0.79-1.11]; p=0.426 |
| Lag7 | 1.02 [0.91-1.15]; p=0.716 | 1.02 [0.96-1.09]; p=0.48 | 0.96 [0.9-1.04]; p=0.34 | 0.95 [0.85-1.06]; p=0.338 |
| Lag14 | 1 [0.83-1.21]; p=0.98 | **0.87 [0.79-0.97]; p=0.008** | **0.89 [0.79-0.99]; p=0.03** | 1.05 [0.9-1.22]; p=0.524 |
| **Atmospheric pressure**  **over 3 days (mean)** | **983 hPa (p_1_)** | **987 hPa (p_5_)** | **1002 hPa (p_95_)** | **1006 hPa (p_99_)** |
| Lag0 | 0.76 [0.43-1.34]; p=0.344 | 1.01 [0.74-1.39]; p=0.95 | 1.11 [0.8-1.53]; p=0.526 | 1.18 [0.67-2.09]; p=0.556 |
| Lag1 | 0.96 [0.39-2.36]; p=0.924 | 0.91 [0.53-1.54]; p=0.716 | 1.1 [0.63-1.91]; p=0.742 | 0.92 [0.34-2.46]; p=0.87 |
| Lag4 | **0.83 [0.69-0.99]; p=0.042** | 0.9 [0.8-1.01]; p=0.076 | 1 [0.89-1.13]; p=0.936 | 0.95 [0.73-1.22]; p=0.672 |
| Lag7 | 0.92 [0.84-1.01]; p=0.07 | **0.89 [0.84-0.95]; p<0.001** | 0.97 [0.9-1.04]; p=0.344 | **0.8 [0.69-0.92]; p=0.002** |
| Lag14 | **0.75 [0.64-0.89]; p<0.001** | 0.99 [0.9-1.09]; p=0.834 | 1.03 [0.93-1.13]; p=0.6 | 1.01 [0.83-1.22]; p=0.952 |

**Suppl. Table 4.** Relative risk for acute otitis media-related EVs after prolonged extreme weather conditions in the months from 1^st^ of April until 30^th^ of September compared to median conditions. Percentiles (1st, 5th, 95th and 99th) for extreme weather conditions were calculated by three-day averaging of mean temperature, relative humidity mean wind-speed or atmospheric pressure or three-day sum in case of precipitation. Brackets contain confidence intervals (95%); Significant results (p≤0.05) are highlighted

| **Relative risk (RR) of AOM-related EVs in Winter months** | | | | |
| --- | --- | --- | --- | --- |
| **Mean temperature** | **-6 °C (p_1_)** | **-2 °C (p_5_)** | **16 °C (p_95_)** | **18 °C (p_99_)** |
| Lag0 | 0.98 [0.41-2.33]; p=0.96 | 0.9 [0.52-1.56]; p=0.71 | 0.87 [0.47-1.62]; p=0.66 | 0.34 [0.09-1.25]; p=0.104 |
| Lag1 | 0.76 [0.21-2.74]; p=0.678 | 0.86 [0.42-1.76]; p=0.676 | 1.06 [0.48-2.35]; p=0.886 | 0.86 [0.19-3.94]; p=0.848 |
| Lag4 | 0.82 [0.59-1.14]; p=0.236 | 0.85 [0.69-1.05]; p=0.136 | 0.95 [0.74-1.22]; p=0.712 | 1.18 [0.73-1.89]; p=0.502 |
| Lag7 | 1.03 [0.86-1.22]; p=0.776 | 1.03 [0.92-1.15]; p=0.614 | 0.94 [0.8-1.09]; p=0.402 | 0.89 [0.65-1.23]; p=0.492 |
| Lag14 | 1.11 [0.84-1.46]; p=0.46 | 1.09 [0.89-1.33]; p=0.394 | 1.25 [0.96-1.61]; p=0.094 | 1.36 [0.99-1.87]; p=0.058 |
| **Relative humidity** | **42 % (p_1_)** | **49 % (p_5_)** | **89 % (p_95_)** | **94 % (p_99_)** |
| Lag0 | 1.14 [0.62-2.1]; p=0.664 | 0.9 [0.64-1.27]; p=0.552 | 0.79 [0.57-1.09]; p=0.144 | 0.97 [0.58-1.61]; p=0.894 |
| Lag1 | 0.72 [0.32-1.65]; p=0.442 | 1.08 [0.76-1.52]; p=0.678 | 1.1 [0.78-1.55]; p=0.594 | 0.84 [0.47-1.51]; p=0.556 |
| Lag4 | 0.95 [0.75-1.22]; p=0.708 | 1.02 [0.86-1.22]; p=0.792 | 0.93 [0.8-1.08]; p=0.354 | 1.07 [0.85-1.34]; p=0.584 |
| Lag7 | 1.06 [0.79-1.41]; p=0.712 | 1 [0.88-1.13]; p=0.978 | 1.06 [0.96-1.18]; p=0.246 | 1.15 [0.99-1.34]; p=0.07 |
| Lag14 | 1.05 [0.66-1.66]; p=0.848 | 1.04 [0.83-1.29]; p=0.742 | 0.88 [0.72-1.06]; p=0.18 | 0.89 [0.66-1.19]; p=0.424 |
| **Precipitation** | - | - | **8 mm (p_95_)** | **17 mm (p_99_)** |
| Lag0 | - | - | 0.99 [0.71-1.38]; p=0.948 | 1.11 [0.41-3.03]; p=0.838 |
| Lag1 | - | - | 0.97 [0.68-1.38]; p=0.848 | 1.62 [0.62-4.24]; p=0.33 |
| Lag4 | - | - | 0.91 [0.74-1.13]; p=0.408 | 1.08 [0.54-2.14]; p=0.828 |
| Lag7 | - | - | 0.97 [0.83-1.13]; p=0.688 | 0.93 [0.54-1.58]; p=0.778 |
| Lag14 | - | - | 0.97 [0.75-1.25]; p=0.818 | 0.93 [0.51-1.7]; p=0.82 |
| **Mean wind speed** | **1 m/s (p_1_)** | **1.5 m/s (p_5_)** | **5.9 m/s (p_95_)** | **7.5 m/s (p_99_)** |
| Lag0 | 0.76 [0.52-1.09]; p=0.138 | 0.86 [0.69-1.08]; p=0.194 | 1.22 [0.91-1.63]; p=0.188 | 0.83 [0.46-1.48]; p=0.526 |
| Lag1 | 0.98 [0.69-1.4]; p=0.914 | 0.95 [0.77-1.18]; p=0.648 | 0.89 [0.64-1.24]; p=0.496 | 1.5 [0.82-2.75]; p=0.194 |
| Lag4 | 0.98 [0.81-1.2]; p=0.88 | 1.01 [0.9-1.14]; p=0.836 | 1.07 [0.89-1.28]; p=0.5 | 1.14 [0.84-1.54]; p=0.404 |
| Lag7 | 0.94 [0.79-1.12]; p=0.508 | 0.99 [0.89-1.09]; p=0.786 | 1.01 [0.87-1.18]; p=0.876 | 0.91 [0.73-1.14]; p=0.424 |
| Lag14 | 0.97 [0.75-1.26]; p=0.824 | 0.98 [0.84-1.15]; p=0.79 | 0.93 [0.74-1.17]; p=0.518 | 1.32 [0.95-1.83]; p=0.1 |
| **Atmospheric pressure** | **974 hPa (p_1_)** | **981 hPa (p_5_)** | **1011 hPa (p_95_)** | **1015 hPa (p_99_)** |
| Lag0 | 1.05 [0.59-1.87]; p=0.862 | 0.87 [0.6-1.26]; p=0.452 | 1.01 [0.68-1.5]; p=0.956 | 0.91 [0.47-1.76]; p=0.786 |
| Lag1 | 1.35 [0.67-2.72]; p=0.4 | 1.32 [0.83-2.08]; p=0.24 | 0.75 [0.45-1.27]; p=0.29 | 0.62 [0.27-1.44]; p=0.268 |
| Lag4 | 0.88 [0.63-1.21]; p=0.42 | 1 [0.85-1.18]; p=0.978 | 0.94 [0.81-1.09]; p=0.398 | 0.93 [0.74-1.17]; p=0.536 |
| Lag7 | 0.84 [0.66-1.07]; p=0.156 | 0.99 [0.89-1.1]; p=0.828 | 1.05 [0.97-1.15]; p=0.232 | 1.02 [0.88-1.18]; p=0.79 |
| Lag14 | 0.99 [0.71-1.37]; p=0.95 | 1.01 [0.83-1.23]; p=0.886 | 0.91 [0.77-1.07]; p=0.248 | **0.74 [0.56-0.97]; p=0.028** |

**Suppl. Table 5.** Relative risk for acute otitis media-related EVs under extreme weather conditions (1st, 5th, 95th and 99th percentile) in the months from 1^st^ of October to 31^st^ of March compared to median conditions. Brackets contain confidence intervals (95%); Significant results (p≤0.05) are highlighted

| **Relative risk (RR) of AOM-related EVs in Winter months**  **after prolonged extreme weather conditions over 3 days** | | | | |
| --- | --- | --- | --- | --- |
| **Mean temperature**  **over 3 days (mean)** | **-5 °C (p_1_)** | **-2 °C (p_5_)** | **16 °C (p_95_)** | **18 °C (p_99_)** |
| Lag0 | 1.51 [0.72-3.17]; p=0.274 | 1.14 [0.73-1.77]; p=0.564 | 1.05 [0.66-1.67]; p=0.836 | 0.8 [0.35-1.82]; p=0.6 |
| Lag1 | 0.77 [0.17-3.36]; p=0.724 | 1.04 [0.45-2.42]; p=0.93 | 0.95 [0.4-2.27]; p=0.916 | 0.95 [0.22-4.03]; p=0.944 |
| Lag4 | 0.91 [0.67-1.26]; p=0.578 | 0.97 [0.8-1.17]; p=0.74 | 0.99 [0.79-1.24]; p=0.91 | 1.06 [0.72-1.55]; p=0.772 |
| Lag7 | 0.99 [0.89-1.11]; p=0.912 | 1 [0.93-1.07]; p=0.908 | 0.97 [0.88-1.06]; p=0.488 | 0.97 [0.82-1.13]; p=0.66 |
| Lag14 | 1.01 [0.88-1.16]; p=0.898 | 1.02 [0.92-1.14]; p=0.644 | **1.21 [1.07-1.38]; p=0.004** | **1.28 [1.08-1.51]; p=0.004** |
| **Relative humidity**  **over 3 days (mean)** | **47 % (p_1_)** | **53 % (p_5_)** | **86 % (p_95_)** | **91 % (p_99_)** |
| Lag0 | 0.9 [0.64-1.26]; p=0.53 | 0.84 [0.67-1.06]; p=0.144 | 0.9 [0.72-1.13]; p=0.376 | 0.97 [0.71-1.33]; p=0.866 |
| Lag1 | 0.87 [0.52-1.46]; p=0.604 | 0.92 [0.67-1.28]; p=0.64 | 0.91 [0.65-1.27]; p=0.578 | 0.88 [0.56-1.37]; p=0.568 |
| Lag4 | 1.15 [1-1.33]; p=0.056 | 1.05 [0.95-1.15]; p=0.322 | **0.9 [0.83-0.98]; p=0.012** | 0.95 [0.85-1.07]; p=0.434 |
| Lag7 | 1.15 [1.03-1.28]; p=0.01 | 1 [0.95-1.06]; p=0.944 | 1.03 [0.98-1.07]; p=0.224 | **1.1 [1.03-1.16]; p=0.002** |
| Lag14 | 1.04 [0.83-1.29]; p=0.736 | 1.06 [0.94-1.18]; p=0.348 | **0.88 [0.81-0.95]; p=0.002** | 0.93 [0.84-1.04]; p=0.206 |
| **Precipitation**  **over 3 days (sum)** | - | - | **18 mm (p_95_)** | **32 mm (p_99_)** |
| Lag0 | - | - | 1.23 [0.98-1.54]; p=0.07 | 0.84 [0.53-1.33]; p=0.462 |
| Lag1 | - | - | 1.14 [0.86-1.52]; p=0.352 | 0.86 [0.5-1.5]; p=0.598 |
| Lag4 | - | - | 1.06 [0.95-1.19]; p=0.264 | 0.77 [0.59-1.01]; p=0.062 |
| Lag7 | - | - | 0.99 [0.93-1.07]; p=0.888 | 1.17 [0.99-1.39]; p=0.07 |
| Lag14 | - | - | 1.1 [0.97-1.24]; p=0.15 | **0.51 [0.35-0.73]; p<0.001** |
| **Mean wind speed**  **over 3 days (mean)** | **1.3 m/s (p_1_)** | **1.9 m/s (p_5_)** | **5.3 m/s (p_95_)** | **6.5 m/s (p_99_)** |
| Lag0 | 0.95 [0.7-1.29]; p=0.758 | 1.03 [0.88-1.22]; p=0.684 | 1 [0.84-1.2]; p=0.964 | 1.04 [0.76-1.42]; p=0.816 |
| Lag1 | 0.88 [0.6-1.29]; p=0.528 | 0.89 [0.72-1.09]; p=0.256 | 1.05 [0.83-1.33]; p=0.688 | 1.15 [0.76-1.75]; p=0.508 |
| Lag4 | 1.08 [0.97-1.21]; p=0.166 | 1.02 [0.96-1.09]; p=0.456 | 1.07 [0.99-1.16]; p=0.09 | 1.09 [0.96-1.23]; p=0.17 |
| Lag7 | 1.01 [0.94-1.09]; p=0.742 | 0.98 [0.94-1.02]; p=0.386 | **0.94 [0.89-0.99]; p=0.026** | 0.97 [0.9-1.05]; p=0.498 |
| Lag14 | **0.85 [0.73-0.98]; p=0.03** | **0.92 [0.86-0.99]; p=0.032** | 0.98 [0.9-1.07]; p=0.61 | 1.07 [0.95-1.21]; p=0.244 |
| **Atmospheric pressure**  **over 3 days (mean)** | **978 hPa (p_1_)** | **983 hPa (p_5_)** | **1010 hPa (p_95_)** | **1013 hPa (p_99_)** |
| Lag0 | 1.11 [0.73-1.7]; p=0.614 | 1.09 [0.8-1.48]; p=0.57 | 0.99 [0.73-1.33]; p=0.922 | 0.9 [0.59-1.39]; p=0.644 |
| Lag1 | 1.13 [0.54-2.36]; p=0.752 | 1.03 [0.59-1.78]; p=0.922 | 0.95 [0.56-1.62]; p=0.844 | 0.93 [0.45-1.93]; p=0.846 |
| Lag4 | 1.1 [0.93-1.3]; p=0.252 | 1.05 [0.94-1.18]; p=0.404 | 0.96 [0.86-1.07]; p=0.468 | 0.96 [0.82-1.11]; p=0.562 |
| Lag7 | 0.93 [0.85-1.01]; p=0.07 | **0.95 [0.9-1]; p=0.038** | 1.02 [0.98-1.07]; p=0.346 | 1.03 [0.96-1.1]; p=0.456 |
| Lag14 | 0.94 [0.82-1.07]; p=0.356 | 1.07 [0.98-1.16]; p=0.118 | 0.97 [0.9-1.05]; p=0.49 | **0.84 [0.76-0.94]; p=0.002** |

**Suppl. Table 6**. Relative risk for acute otitis media-related EVs after prolonged extreme weather conditions in the months from 1^st^ of October to 31^st^ of March compared to median conditions. Percentiles (1st, 5th, 95th and 99th) for extreme weather conditions were calculated by three-day averaging of mean temperature, relative humidity mean wind-speed or atmospheric pressure or three-day sum in case of precipitation. Brackets contain confidence intervals (95%); Significant results (p≤0.05) are highlighted
