# Supplementary material for: Fluctuations in emergency department visits related to acute otitis media are associated with extreme meteorological conditions
Source: Front Public Health. 2023 Jun 1;11:1153111. doi: 10.3389/fpubh.2023.1153111 (PMC10267338; doi:10.3389/fpubh.2023.1153111)
Supplement: Supplementary file 2 [file Image_1.pdf]

Supplementary Figures

Fluctuations in emergency department visits related to Acute otitis media are associated with extreme meteorological conditions

Michael Nieratschker, MD[1], Markus Haas, BA[1], Mateo Lucic[1], Franziska Pichler[1], Faris F. Brkic, MD PhD[1], Thomas Parzefall, MD PhD[1], Dominik Riss, MD[1*], David T. Liu, MD PhD[1]

1 Department of Otorhinolaryngology, Head and Neck Surgery, Medical University of Vienna, Vienna, Austria

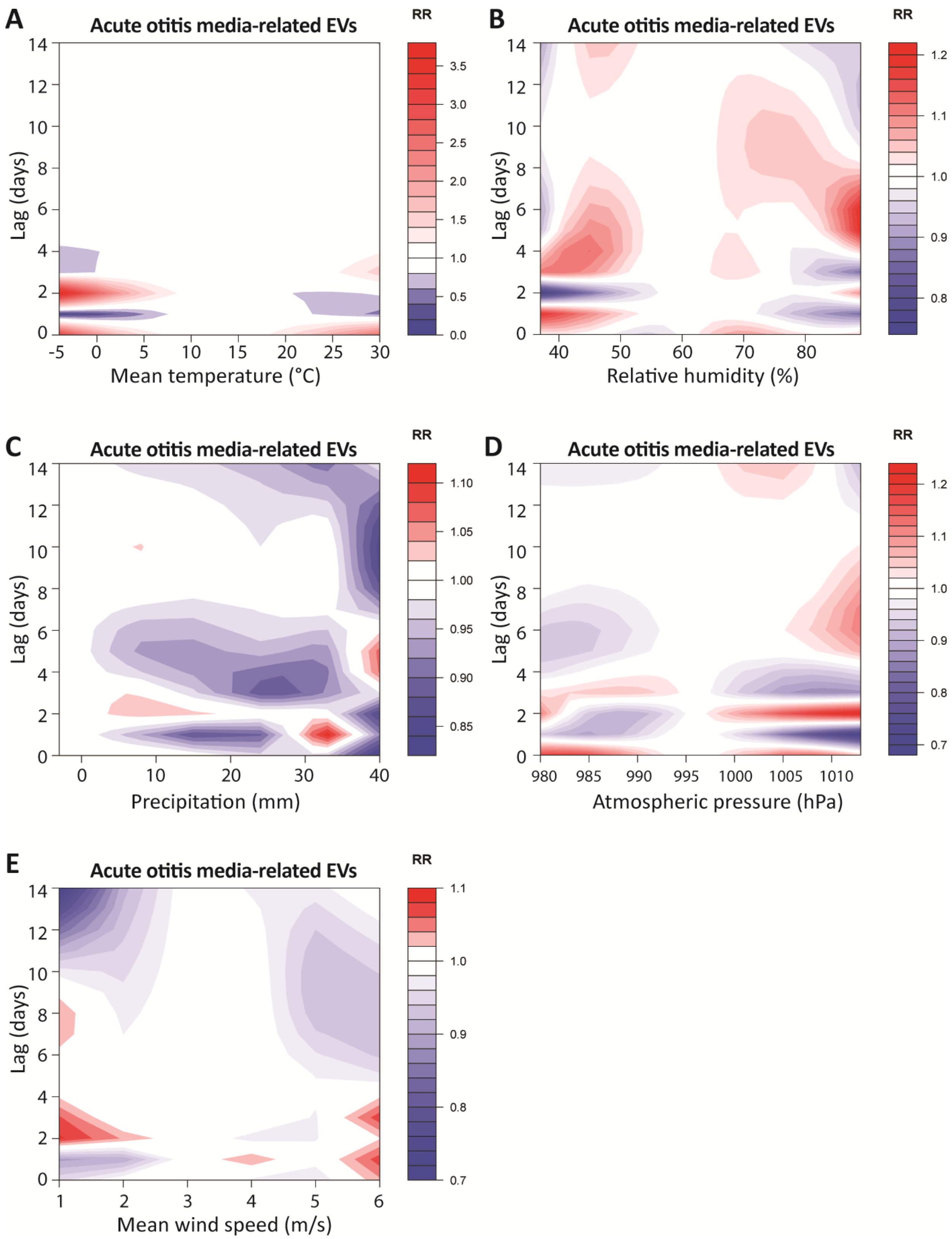

Suppl. Fig 1. Contour-plots of relative risk for acute otitis media-related EVs after sustained extreme weather events of three days of mean temperature in °C (**A**), mean relative humidity in % (**B**), sum of precipitation in mm (**C**), mean atmospheric pressure in hPa (**D**) and mean wind speed in m/s (**E**) from lag0 to lag14
